# Data on possible in vitro anti-diabetic effects of verticinone on β-TC6 pancreatic and C2C12 skeletal muscle cells

**DOI:** 10.1016/j.dib.2019.104828

**Published:** 2019-11-26

**Authors:** Fargol Mashhadi Akbar Boojar, Reza Aghaei, Mahdi Mashhadi Akbar Boojar

**Affiliations:** a Faculty of Dentistry, University of Golestan Medical Science, Gorgan, Iran; b Islamic Azad University Shabestar Branch, Islamic Azad University, Shabestar, Iran; c Department of Pharmacology and Toxicology, Faculty of Pharmacy, Baqiyatallah University of Medical Sciences, Tehran, Iran; d Student Research Committee, Baqiyatallah University of Medical Sciences, Tehran, Iran

**Keywords:** Advanced glycation end product, Glucose uptake, Glyoxalase, Insulin secretion, Verticinone

## Abstract

Verticinone as a steroidal alkaloid is one of the major active constituents of medicinal herb, *Fritillaria imperialis* with several pharmacological properties. Present data demonstrate an in vitro assessment of verticinone effects on β-TC6 pancreatic and C2C12 skeletal muscle cells include cell survival, activities of carbohydrate-hydrolyzing enzymes (α-amylase and α-glucosidase), levels of insulin secreted into the media, glucose uptake ability, advanced glycation end product (AGEs) include 3-deoxyglucosone, methylglyoxal, and pentosidine levels and the activity of glyoxalase I. Data reveals possible hypoglycemic potential of verticinone, although, the high concentrations of this compound were associated with elevated amount of AGEs and it should be assessed in future investigations.

**Table:** Specifications Table

Subject area	Biology
Specific subject area	The advanced glycation end product, Glucose uptake, Insulin secretion, carbohydrate-hydrolyzing enzymes activities, *Fritillaria imperialis* hypoglycemic effects
Type of data	Tables and graphs
How data was acquired	Multi-Label Reader (Hidex, Turku, Finland), ArrayScan high content screening system (Cellomics Inc., Pittsburgh, PA, USA), C18 reversed-phase separation column (Nova-Pak, 150 × 3 mm) and TSK-GEL ODS-80TM column of HPLC system
Data format	Raw and analyzed data
Parameters for data collection	β-TC6 pancreatic and C2C12 skeletal muscle cells were exposed to verticinone.
Description of data collection	Two independent cells were treated to various concentrations of verticinone and after 24 hours of incubation, they were prepared and evaluated using the biological assays based on UV/VIS spectrophotometric and HPLC methods.
Data source location	Faculty of Pharmacy, Baqiyatallah University of Medical Sciences, Tehran, Tehran Province, Iran.
Data accessibility	Raw data are available within this article as supplementary material.

**Table:** 

**Value of the Data** • Data including verticinone hypoglycemic effects may be of value to the researchers working on the treatment of diabetes using natural medicines. • Data showing that hypoglycemic effects of verticinone are due to increased insulin secretion and glucose uptake and inhibition of carbohydrate-hydrolyzing enzymes may be of potential value for the scientists working on biological mechanisms of phytomedicine in diabetes mellitus. • Data showing that verticinone high doses can lead to increased production of toxic glycation intermediates may be useful for the researchers working on drug discovery and herbal medicine.

## 1 Data

Verticinone ((1R, 2S, 6S, 9S, 11S, 14S, 15S, 18S, 20S, 23R, 24S)-10, 20-dihydroxy-6, 10, 23-trimethyl-4-azahexacyclo [12.11.0.02, 11.04, 9.015, 24.018, 23] pentacosan-17-one) is a widely known steroidal alkaloid with several pharmacological properties and it is regarded as one of the major active constituents of medicinal herb, *Fritillaria imperialis* [[1], [2], [3]]. However, this compound has never been evaluated in vitro for hypoglycemic and possible anti-diabetes activities.

**Fig. 1:**
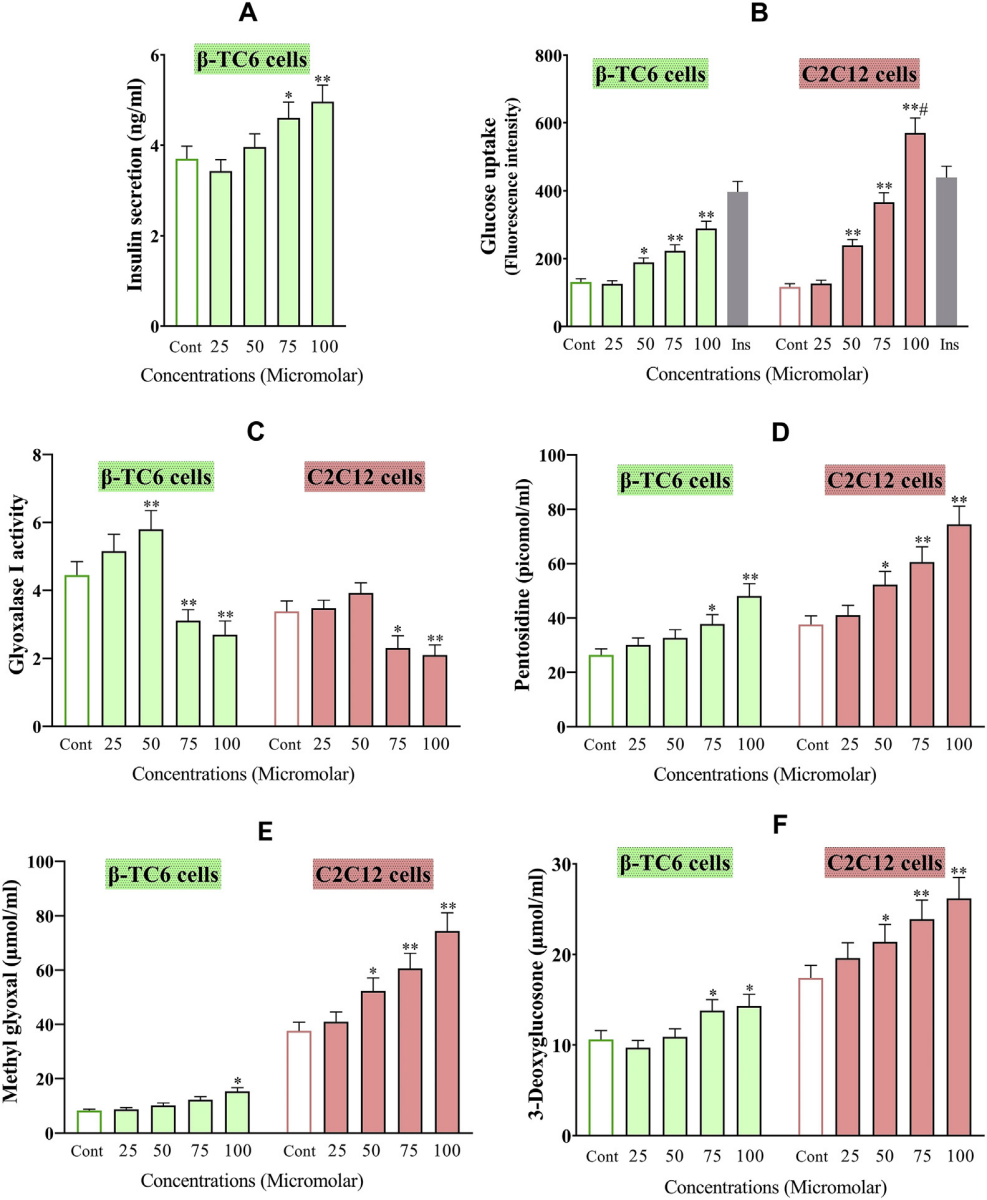
**A:** Insulin secretion levels of β-TC6 pancreatic cells, **B:** The glucose uptake as fluorescence intensity of 2NBDG, **C:** The activities of glyoxalase I, and **D:** The content of Pentosidine (picomol/mL), **E:** Methylglyoxal (μmol/mL), and **F:** 3-Deoxyglucosone (μmol/mL) in the extract of C2C12 and β-TC6 cells after 24 h incubation with different concentrations of verticinone. All biological assays were performed separately in cell lysate samples and data presented as mean ± SD; N = 3. *Significant difference at P < 0.05 compared to the control group (normal saline) according to one-way ANOVA, followed by Tukey's post hoc test. ** Significant difference at P < 0.001 in comparison to control.

Current data is about verticinone effects on β-TC6 pancreatic and C2C12 skeletal muscle cells. The cytotoxicity of verticinone against β-TC6 and C2C12 cells and 50% cell mortality (IC50) for the assessed compound and doxorubicin (as a standard cytotoxic agent) expressed in Table 1, Table 2. Table 3 shows the half-maximal effective concentration (EC50) of verticinone and Acarbose (as a standard inhibitor) on α-glucosidase and α-amylase activities. Verticinone effects on β-TC6 cells insulin secretion and glucose uptake, glyoxalase I activities and AGEs (Pentosidine, Methylglyoxal, and 3-Deoxyglucosone) of β-TC6 and C2C12 cells were presented in Fig. 1. The raw data file is included as supplementary material in this article.

**Table 1:** The cell viability of C2C12 and β-TC6 cells (percent of control) after 24 h incubation with different concentrations of verticinone assessed by the MTT assay. The cytotoxic response of the investigated compound at each concentration was analyzed separately in independent cell lysate samples. Data are expressed as mean survival relative to the untreated control ± SD; N = 3.

Cells	Control	Verticinone (μg/mL)			
		25	50	75	100
C2C12	100 ± 5.2	77.0 ± 6.4	51.5 ± 4.4	38.6 ± 3.0	19.6 ± 1.3
β-TC6	100 ± 5.0	71.6 ± 6.0	47.8 ± 3.8	33.5 ± 2.7	17.8 ± 1.5

**Table 2:** The cytotoxicity (IC50) of β-TC6 and C2C12 under treatment with different concentrations of verticinone and doxorubicin.

Cells	Doxorubicin (μg/mL)	Verticinone (μg/mL)
β-TC6	4.1	48.4
C2C12	3.5	44.2

**Table 3:** The inhibitory effect verticinone on α-amylase and α-glucosidase activities based on IC50 (dosage that inhibited 50% of enzyme activity) values. Acarbose considered as a positive standard.

Enzyme	Compound	IC50 (μg/mL)
α-Amylase	Verticinone	62
	Acarbose	30
α-Glucosidase	Verticinone	155
	Acarbose	170

Data reveals the possible anti-diabetic potential of verticinone, although, the high concentrations of this compound were associated with elevated levels of AGEs and it should be assessed in future investigations.

## 2 Experimental design, materials, and methods

### 2.1 Reagents, cell line, and cell cultures

Mouse myoblast (skeletal muscle) C2C12 and β-TC6 pancreatic cells were purchased from Institute Pasteur Medical Center (Tehran, Iran). Verticinone standard (purity>98.0%) were purchased from Sigma-Aldrich, St. Louis, MO and other chemicals and reagents were obtained from Gibco Laboratory (Invitrogen Co, Grand Island, NY, USA). The cells were grown in Dulbecco's modified Eagle's medium (Life Technologies, Inc., Rockville, MD) supplemented with heat-inactivated fetal bovine serum (FBS), penicillin/streptomycin antibiotics, l-glutamine and HEPES Na and were cultured in a humidified incubator at 37 °C with 5% of CO_2_ [4].

Each experiment began with the preparation of 1 × 10^6^ cells/mL suspension and the samples treated independently with increasing concentrations of verticinone (0, 25, 50, 75 and 100, micromolar) for 24 hours. The tests were repeated three times separately.

### 2.2 Cell viability

The assessment of cell viability was performed according to the 3-(4,5-dimethylthiazol-2-yl)-2,5-diphenyl tetrazolium bromide (MTT) colorimetric assay. After independent treatment of the C2C12 and β-TC6 cells with verticinone, cells were washed and then incubated with MTT solution to reach the desired final concentration and after 3 h, dimethyl sulfoxide (DMSO) added. The absorbance ratio of the verticinone treated cells to the absorbance of DMSO-treated control cells was determined using spectrophotometrically at 570 nm (UV-VIS) by the Multi-Label Reader (Hidex, Turku, Finland). The obtained data were expressed as survival percentages in comparison to control [5,6].

### 2.3 α-Amylase activity assay

After the incubation period, the cell suspensions, sodium phosphate buffer and high purity α-amylase solutions were incubated at room temperature for 10 min. Then a starch solution in sodium phosphate buffer was added. After 10 minutes, the reaction was stopped with dinitrosalicylic acid reagent. The obtained solution was then placed in boiling water (in Ben Mari) and cooled to 25 °C. Acarbose considered as a positive standard and finally, the absorbance of reaction mixtures was measured at 540 nm (UV-VIS) using multimode-reader [7].

### 2.4 α-Glucosidase activity assay

Briefly, α-glucosidase from *Saccharomyces cerevisiae* was added to cell suspensions and the obtained mixture incubated with phosphate buffer solution for 5 min at 37 °C. Then para-nitrophenyl-α-D glucopyranoside in phosphate buffer was added and mixed to initiate the reaction. Acarbose was used as a positive control again. Finally, the reaction was stopped by the addition of Na_2_CO_3_ and the absorbance was determined at 405 nm [8].

### 2.5 Insulin secretion assay

To quantifying insulin secretion, the β-TC6 pancreatic cell line was grown in RPMI media containing glucose, FBS, penicillin, and streptomycin. After exposure of β-TC6 cells to different concentrations of verticinone, the cells were washed and incubated in Krebs-Ringers bicarbonate (KRB) buffer and glucose. After incubation and centrifugation, the aliquots of supernatants were stored at −20 °C until the final experiment (insulin assessment). The mouse insulin ELISA kit (Shibayagi Co.) was used to determine insulin levels [9].

### 2.6 Glucose uptake assay

The overnight incubation of test cells at 96-well plate was done, the cell suspensions washed and refilled with 2.5 mM solution of glucose and DMEM supplemented with l-glutamine and FBS. After a period of pre-incubation, the medium was replaced with 2-[N-(7-nitrobenz-2-oxa-1,3-diazol-4-yl)amino]-2-deoxy-d-glucose (2-NBDG). In the following, 2-NBDG endocytosed to the cells and then the medium was discarded, cells were washed with PBS and stained with dye Hoechst 33342. Finally, the fluorescence intensity of 2-NBDG determined at 350/461 nm using the ArrayScan high content screening system (Cellomics Inc., Pittsburgh, PA, USA) [10].

### 2.7 Glyoxalase-1 activity assay

The assessment of glyoxalase-1 activity was performed using a spectrophotometric method which determined the absorbance of S-d-lactoylglutathione at 240 nm. The standard assay solution contained methylglyoxal, glutathione, magnesium sulfate, and phosphate potassium. The reaction initiated by adding the kidney extract to the test mixture for hemithioacetal formation. One unit of activity was expressed as the generation of 1 mM of S-d-lactoylglutathione/min/mg protein of cell extract [11].

### 2.8 Methylglyoxal assay

For determination of methylglyoxal, the supernatant of cell cultures added to water and phosphate buffer supplemented with 4-Methoxy-o-phenylenediamine (4MPD). The obtained solution was incubated, acidified with HCl, diluted with acetonitrile, saturated with NaCl and centrifuged. The acetonitrile layer injected into an HPLC-FLD (fluorimetric detector) system. Separation of methylglyoxal was accomplished by three mobile phases: A [water], B [acetonitrile], and C [acetic acid and triethanolamine]. Fluorimetric detection performed using excitation and emission wavelengths at 344 and 420 nm, respectively [12].

### 2.9 Pentosidine assay

To determine the pentosidine levels, the samples were diluted 1: 1000 with phosphate buffer and incubated for an overnight period. Then, the remained mixture was concentrated by evaporator and hydrolyzed by adding HCl. Hydrochloric acid was also evaporated again by an evaporator and then diluted by water and neutralized by the addition of sodium hydroxide. The obtained solution was used for injection to C18 reversed-phase separation column (Nova-Pak, 150 × 3 mm) in the HPLC-FL system. The pentosidine was detected by fluorescence at 325/385 nm [13].

### 2.10 3-Deoxyglucosone (3-DG) assay

The quantifying method for evaluation of 3-DG levels was on the basis of HPLC separation and UV detection. Briefly, the cell extract or 3-DG standard added to the perchloric acid solution and centrifuged. Afterward, disodium carbonate added to neutralizing the supernatant and then 2, 3-diaminonaphthalene and 2,3-pentanedione added to obtained mixture. After extraction using ethyl acetate, methanolic solution of the dried extract was injected to reverse phase TSK-GEL ODS-80TM column of HPLC system. Finally, 3-DG levels were determined using a UV detector at 280 nm [14].

### 2.11 Statistical analysis

Data expressed as mean ± S.D. in 5 groups (triplicate) and analyzed with one-way analysis of variance (ANOVA) followed by Tukey's post hoc test using SPSS software (version 19.0) to demonstrate the statistical difference. A P value of less than 0.05 was considered significant. The IC50 values were determined by GraphPad Prism software (version 8.0) and the graphs were drawn.

## Conflict of Interest

The authors declare that they have no known competing financial interests or personal relationships that could have appeared to influence the work reported in this paper.
